# Supplementary figures and images for: Identification of French Guiana anopheline mosquitoes by MALDI-TOF MS profiling using protein signatures from two body parts
Source: PLoS One. 2020 Aug 20;15(8):e0234098. doi: 10.1371/journal.pone.0234098 (PMC7444543; doi:10.1371/journal.pone.0234098)

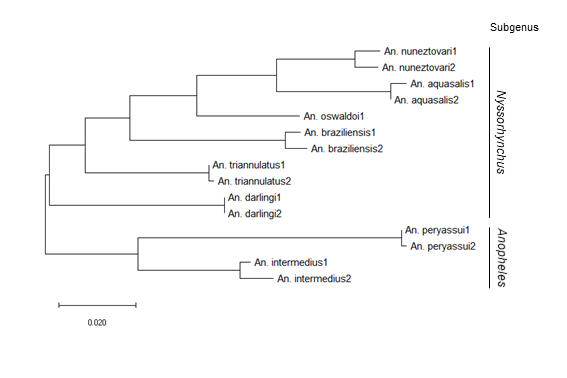

Supplement: S1 Fig — (TIF) [file pone.0234098.s001.tif]

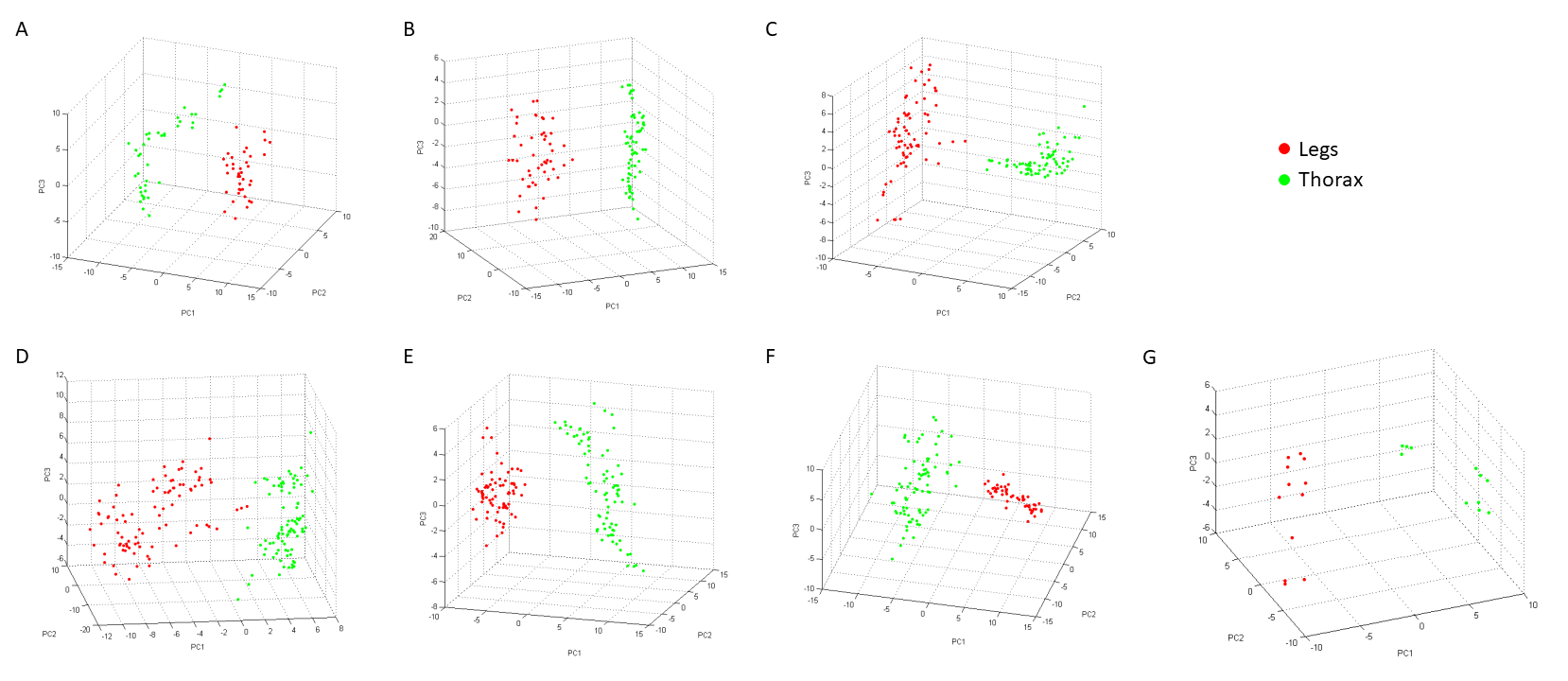

Supplement: S2 Fig — PCA dimensional image from MS spectra of legs (red dots) and thoraxes (green dots) from An. intermedius (A), An. aquasalis (B), An. braziliensis (C), An. darlingi (D), An. nuneztovari (E), An. triannulatus (F) and An. peryassui (G). Respectively, 10, 22, 18, 22, 20, 20 and 3 specimens per species were included. Quadruplicate of each sample per body part were presented. (TIF) [file pone.0234098.s002.tif]

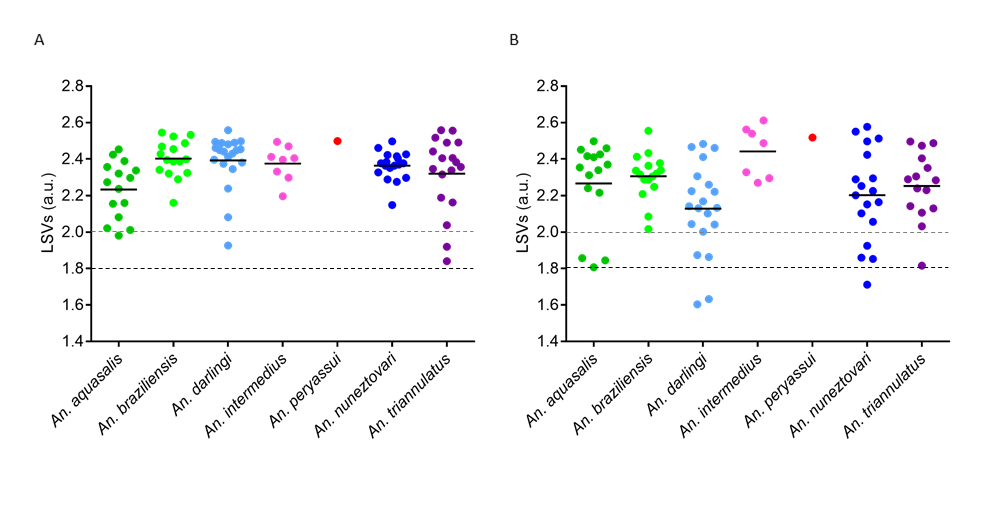

Supplement: S3 Fig — LSVs obtained following homemade MS reference database query with MS spectra of legs (A) and thoraxes (B) from Anopheles mosquitoes. Horizontal dashed lines represent the threshold value for reliable identification (black and grey for LSV threshold of 1.8 and 2.0, respectively). LSVs, log score values; a.u., arbitrary units. (TIF) [file pone.0234098.s003.tif]
