# Supplementary material for: Identification of French Guiana anopheline mosquitoes by MALDI-TOF MS profiling using protein signatures from two body parts
Source: PLoS One. 2020 Aug 20;15(8):e0234098. doi: 10.1371/journal.pone.0234098 (PMC7444543; doi:10.1371/journal.pone.0234098)
Supplement: S1 Table — (DOCX) [file pone.0234098.s004.docx]

**S1 Table.** Top-ten and top-five mass peak list per mosquito species using legs as biologic material.

|  |  | Average peak intensity (a.u.)* | | | | | | | |
| --- | --- | --- | --- | --- | --- | --- | --- | --- | --- |
| **MS peak number§** | **m/z (Da)** | ***An. intermedius*** | ***An. minor*** | ***An. aquasalis*** | ***An. braziliensis*** | ***An. darlingi*** | ***An. nuneztovari*** | ***An. oswaldoi*** | ***An. triannulatus*** |
| 1 | 2004.8 | 2.4 | 2.9 | 4.4 | 1.8 | **9.9** | 1.9 | 2.4 | 3.4 |
| 2 | 2615.8 | 3.8 | 2.7 | 4.3 | 4.3 | 3.5 | 2.0 | 2.7 | **14.5** |
| 3 | 2623.3 | ***15.4*** | 1.9 | 4.1 | 1.6 | 2.1 | 4.3 | 1.8 | 4.0 |
| 4 | 2630.3 | 3.0 | 1.8 | **10.2** | 1.4 | 2.7 | 2.1 | 2.3 | 3.0 |
| 5 | 4184.3 | 1.9 | 1.9 | 1.8 | 1.0 | 2.0 | 1.9 | ***19.3*** | 1.7 |
| 6 | 4283.9 | 5.8 | 3.7 | 3.1 | 6.1 | **8.3** | 3.8 | 3.6 | 4.3 |
| 7 | 4520.8 | 4.7 | ***23.3*** | 2.8 | 4.9 | 3.0 | 2.0 | 1.5 | 1.8 |
| 8 | 4593.9 | 5.3 | 6.5 | ***18.0*** | 1.6 | 2.9 | 2.4 | 2.8 | 2.2 |
| 9 | 4620.0 | 3.9 | ***58.1*** | 5.6 | 2.5 | 7.2 | 3.4 | 3.9 | 3.5 |
| 10 | 4731.9 | 1.6 | 1.6 | 2.3 | 1.6 | ***41.3*** | 2.0 | 1.7 | 4.6 |
| 11 | 4838.0 | 1.2 | 1.0 | 3.4 | ***14.9*** | ***14.2*** | 2.6 | 1.2 | ***33.5*** |
| 12 | 4854.1 | 2.3 | 1.1 | 8.1 | 2.5 | 2.9 | ***14.8*** | 1.4 | 4.1 |
| 13 | 4868.0 | 1.6 | 1.2 | ***25.2*** | 1.8 | 2.6 | 2.4 | 1.3 | 4.0 |
| 14 | 5025.8 | 2.2 | ***16.9*** | 2.0 | 4.1 | 1.7 | 7.3 | **9.4** | 5.7 |
| 15 | 5060.8 | ***19.2*** | 5.9 | 3.1 | 1.5 | 4.2 | 1.5 | 1.4 | 1.7 |
| 16 | 5132.5 | 3.7 | 5.7 | 3.1 | 1.9 | 4.3 | **10.5** | 4.3 | **9.1** |
| 17 | 5179.2 | 3.8 | ***78.8*** | 2.4 | 2.5 | 3.6 | 1.8 | 3.9 | 3.0 |
| 18 | 5208.6 | ***18.9*** | **9.4** | 2.7 | **8.6** | 4.0 | 3.0 | 2.0 | **9.9** |
| 19 | 5225.8 | **13.9** | **9.0** | 4.0 | 7.3 | **10.9** | 7.9 | 7.0 | **10.1** |
| 20 | 5237.7 | **11.2** | ***10.6*** | **13.1** | ***27.3*** | ***17.1*** | **8.2** | 5.1 | ***97.1*** |
| 21 | 5253.2 | ***137.1*** | 6.3 | ***18.9*** | 7.8 | 4.7 | ***32.9*** | 2.9 | ***15.7*** |
| 22 | 5267.2 | **13.1** | 4.5 | ***56.5*** | 3.4 | 2.6 | 5.8 | 3.2 | 6.9 |
| 23 | 5293.1 | 9.0 | **7.7** | **9.5** | 2.5 | 2.1 | 3.0 | ***23.2*** | 5.0 |
| 24 | 5348.3 | **9.6** | **9.6** | 8.5 | 4.2 | 4.8 | 7.2 | ***10.6*** | 6.4 |
| 25 | 5403.9 | **13.2** | 4.9 | ***23.8*** | 1.3 | 1.8 | ***35.8*** | ***22.0*** | **10.6** |
| 26 | 5428.8 | ***15.1*** | 2.0 | **9.3** | 1.1 | 5.5 | 8.0 | 7.2 | ***51.0*** |
| 27 | 5464.7 | 4.4 | 1.2 | 8.1 | 1.2 | ***26.5*** | 6.9 | 6.3 | 8.0 |
| 28 | 5474.5 | 8.2 | 1.3 | 8.3 | 1.2 | **13.3** | 7.6 | **7.6** | 7.2 |
| 29 | 5663.7 | 6.8 | 2.2 | 3.8 | **12.5** | 4.7 | 4.7 | 4.2 | 2.2 |
| 30 | 5672.8 | 2.6 | 1.6 | 2.0 | ***18.6*** | 3.6 | 2.8 | 3.8 | 1.4 |
| 31 | 5685.6 | 3.4 | 1.7 | 1.7 | ***38.0*** | 3.7 | 2.1 | 2.5 | 1.7 |
| 32 | 5853.7 | 6.9 | **8.4** | 1.3 | 5.9 | 1.0 | 1.1 | 0.9 | 0.9 |
| 33 | 10054.4 | 3.2 | 1.5 | 2.1 | 3.2 | 0.6 | **9.0** | **9.0** | 3.8 |
| 34 | 10803.8 | 2.5 | 2.2 | **16.7** | 0.4 | 2.1 | ***36.7*** | ***19.4*** | 7.2 |
| 35 | 10852.3 | 8.5 | 1.4 | 5.6 | 0.4 | 5.6 | ***10.8*** | **9.5** | ***34.6*** |
| 36 | 10926.0 | 2.9 | 1.1 | 6.0 | 0.8 | ***24.8*** | **9.1** | **8.5** | 7.4 |
| 37 | 11001.2 | 2.6 | 0.9 | 5.5 | 0.4 | **8.7** | **8.5** | 7.0 | 7.6 |
| 38 | 11365.1 | 2.0 | 1.5 | 2.7 | ***27.4*** | 3.7 | 4.5 | 3.1 | 3.1 |
| 39 | 11460.1 | 0.8 | 5.4 | 1.2 | **8.6** | 1.7 | 1.6 | 1.3 | 1.8 |
| 40 | 11521.6 | 0.5 | 2.0 | 0.9 | **13.2** | 1.2 | 0.9 | 0.9 | 1.1 |
| 41 | 11745.7 | 1.8 | 2.9 | 0.4 | **8.7** | 0.3 | 0.5 | 0.5 | 0.4 |

§List of MS peaks used to distinct *Anopheles* species based on the Genetic Algorithm model analysis of ClinProTools. *The top-ten mass peaks per *Anopheles* species are indicated in bold. Top-five mass peak list per mosquito species are indicated in italic and bold. Da: Daltons; m/z: mass to charge; a.u.: arbitrary unit.
