# Supplementary material for: Identification of French Guiana anopheline mosquitoes by MALDI-TOF MS profiling using protein signatures from two body parts
Source: PLoS One. 2020 Aug 20;15(8):e0234098. doi: 10.1371/journal.pone.0234098 (PMC7444543; doi:10.1371/journal.pone.0234098)
Supplement: S2 Table — (DOCX) [file pone.0234098.s005.docx]

**S2 Table.** Top-ten mass peak list per mosquito species using thoraxes as biologic material.

|  |  | Average peak intensity (a.u.)* | | | | | | | |
| --- | --- | --- | --- | --- | --- | --- | --- | --- | --- |
| **MS peak number§** | **m/z (Da)** | ***An. intermedius*** | ***An. peryassui*** | ***An. aquasalis*** | ***An. braziliensis*** | ***An. darlingi*** | ***An. nuneztovari*** | ***An. oswaldoi*** | ***An. triannulatus*** |
| 1 | 2004.9 | 1.6 | 5.9 | 7.2 | 4.0 | **9.8** | 1.8 | 4.7 | 1.8 |
| 2 | 2018.5 | 1.4 | 11.9 | **16.9** | **12.6** | ***33.2*** | 1.6 | 7.9 | 1.9 |
| 3 | 2134.5 | 1.0 | 3.7 | 2.2 | 6.0 | 4.0 | 1.3 | **17.4** | 1.8 |
| 4 | 2263.7 | 1.0 | 1.6 | 2.5 | 4.9 | 2.8 | 1.2 | ***20.6*** | 1.7 |
| 5 | 3153.5 | 3.1 | 1.8 | ***20.8*** | ***21.8*** | **10.2** | ***32.5*** | 4.7 | **13.4** |
| 6 | 3168.9 | 6.1 | 1.8 | 4.3 | 4.8 | 2.6 | 4.7 | ***18.8*** | 5.8 |
| 7 | 3173.3 | ***26.9*** | 4.5 | 3.0 | 4.3 | 2.1 | 2.8 | **13.4** | 4.4 |
| 8 | 3184.9 | 4.6 | ***26.7*** | 2.9 | 3.8 | 2.3 | 2.3 | 4.3 | 2.8 |
| 9 | 3204.9 | 2.2 | 2.5 | **14.5** | **13.2** | 6.4 | 10.1 | 5.2 | **11.7** |
| 10 | 3398.0 | 11.4 | 11.3 | 7.3 | ***20.5*** | 7.8 | 7.6 | 7.6 | 2.2 |
| 11 | 4339.1 | 10.9 | **14.9** | 8.1 | 11.8 | 6.6 | **17.7** | 12.6 | 6.7 |
| 12 | 4394.0 | 2.1 | 4.0 | 2.2 | 3.5 | 2.5 | **20.1** | 2.4 | 4.2 |
| 13 | 4436.7 | **16.4** | 14.6 | 11.7 | 7.8 | 3.3 | 3.6 | **14.1** | 3.3 |
| 14 | 4476.5 | 3.4 | 3.6 | 2.2 | 2.3 | 1.6 | **12.0** | 1.1 | 1.3 |
| 15 | 4494.1 | 3.1 | **17.8** | 2.4 | 4.2 | 1.3 | 2.8 | 1.5 | 1.2 |
| 16 | 4509.6 | 6.6 | 8.8 | 3.8 | ***33.6*** | 1.6 | 3.7 | 1.5 | 2.1 |
| 17 | 4535.6 | 2.2 | 4.1 | ***20.1*** | 3.3 | 4.7 | 10.2 | 1.8 | 7.0 |
| 18 | 4591.9 | 1.9 | 1.7 | **14.5** | 1.3 | 1.6 | 1.9 | 0.7 | 1.8 |
| 19 | 4730.5 | 1.0 | 0.8 | 1.2 | 3.9 | ***13.9*** | 1.5 | 1.7 | 1.7 |
| 20 | 5060.8 | ***39.0*** | 3.7 | 1.2 | 3.8 | 1.3 | 0.6 | 1.3 | 0.7 |
| 21 | 5105.3 | 3.2 | 2.9 | ***33.1*** | 0.8 | 6.0 | 1.9 | 1.0 | 1.9 |
| 22 | 5117.7 | 3.0 | 1.9 | 8.2 | 0.6 | ***18.7*** | 3.2 | 0.9 | 2.8 |
| 23 | 5132.2 | 5.9 | 3.7 | 3.1 | 1.0 | 6.1 | **24.1** | 2.3 | ***33.9*** |
| 24 | 5208.2 | ***38.1*** | 3.0 | 2.0 | 7.1 | 4.5 | 4.4 | 5.6 | 3.8 |
| 25 | 5226.3 | 6.5 | 10.2 | 4.8 | **13.7** | ***21.5*** | ***27.9*** | ***19.8*** | ***14.9*** |
| 26 | 5240.7 | 3.6 | ***30.5*** | **12.6** | 5.7 | **9.6** | 10.0 | 8.4 | ***22.6*** |
| 27 | 5347.9 | 10.0 | 3.7 | 2.3 | 2.4 | 5.3 | 9.6 | 4.1 | **13.6** |
| 28 | 6124.7 | **15.2** | **18.0** | 5.4 | 5.0 | 7.3 | 11.1 | 11.0 | 3.4 |
| 29 | 6311.9 | 11.8 | 5.8 | ***42.9*** | ***44.9*** | ***28.0*** | ***70.1*** | **14.7** | ***27.2*** |
| 30 | 6342.5 | **20.7** | 9.1 | 10.1 | 7.8 | 7.4 | 10.8 | ***34.3*** | ***17.7*** |
| 31 | 6351.6 | ***60.5*** | ***20.3*** | 9.5 | 7.3 | 6.5 | 9.2 | ***25.7*** | **12.6** |
| 32 | 6371.6 | 12.7 | ***51.0*** | 8.5 | 6.4 | 5.8 | 8.1 | 6.5 | 6.9 |
| 33 | 7265.4 | 8.1 | 0.8 | 8.6 | 5.6 | 5.8 | **14.1** | 5.9 | 5.6 |
| 34 | 8183.9 | **16.1** | 1.0 | 1.4 | 1.3 | 1.7 | 4.5 | 6.8 | 2.5 |
| 35 | 8270.3 | 3.4 | **16.3** | 9.4 | **12.4** | 9.0 | 10.3 | 5.1 | 8.7 |
| 36 | 8676.6 | **19.1** | ***21.9*** | **12.8** | ***14.7*** | **11.2** | ***26.7*** | **15.2** | **10.7** |
| 37 | 8787.8 | 4.2 | 2.9 | 4.7 | 2.7 | 4.5 | ***29.6*** | 1.8 | 8.9 |
| 38 | 8832.9 | 6.7 | 4.1 | 6.9 | 2.2 | **11.9** | 9.8 | 4.1 | 10.1 |
| 39 | 8874.7 | ***21.2*** | **17.4** | ***18.3*** | **11.8** | 7.1 | 5.6 | 12.8 | 6.4 |

§List of MS peaks used to distinct *Anopheles* species based on the Genetic Algorithm model analysis of ClinProTools. *The top-ten mass peaks per *Anopheles* species are indicated in bold. Top-five mass peak list per mosquito species are indicated in italic and bold. Da: Daltons; m/z: mass to charge; a.u.: arbitrary unit.
